# Prevalence and risk factors associated with *Leishmania* infection in Trang Province, southern Thailand

**DOI:** 10.1371/journal.pntd.0006095

**Published:** 2017-11-20

**Authors:** Jipada Manomat, Saovanee Leelayoova, Lertwut Bualert, Peerapan Tan-ariya, Suradej Siripattanapipong, Mathirut Mungthin, Tawee Naaglor, Phunlerd Piyaraj

**Affiliations:** 1 Department of Microbiology, Faculty of Science, Mahidol University, Bangkok, Thailand; 2 Department of Parasitology, Phramongkutklao College of Medicine, Bangkok, Thailand; 3 Department of Medicine, Trang Hospital, Trang, Thailand; National Institutes of Health, UNITED STATES

## Abstract

**Background:**

Autochthonous cutaneous and visceral leishmaniasis (VL) caused by *Leishmania martiniquensis* and *Leishmania siamensis* have been considered emerging infectious diseases in Thailand. The disease burden is significantly underestimated, especially the prevalence of *Leishmania* infection among HIV-positive patients.

**Methods:**

A cross-sectional study was conducted to determine the prevalence and risk factors associated with *Leishmania* infection among patients with HIV/AIDS living in Trang province, southern Thailand, between 2015 and 2016. Antibodies against *Leishmania* infection were assayed using the direct agglutination test (DAT). DNA of *Leishmania* was detected by ITS1-PCR using the buffy coat. Species of *Leishmania* were also identified.

**Results:**

Of 724 participants, the prevalence of *Leishmania* infection was 25.1% (182/724) using either DAT or PCR assays. Seroprevalence of *Leishmania* infection was 18.5% (134/724), while *Leishmania* DNA detected by the PCR method was 8.4% (61/724). Of these, 24.9% (180/724) were asymptomatic, whereas 0.3% (2/724) were symptomatic VL and VL/CL (cutaneous leishmaniasis). At least five species were identified: *L. siamensis*, *L*. *martiniquensis*, *L*. *donovani* complex, *L*. *lainsoni*, and *L*. *major*. Multivariate analysis showed that CD4^+^ levels <500 cells/μL and living in stilt houses were independently associated with *Leishmania* infection. Those who were PCR positive for *Leishmania* DNA were significantly associated with a detectable viral load, whereas non-injection drug use (NIDU) and CD4^+^ levels <500 cells/μL were potential risk factors of *Leishmania* seropositivity.

**Conclusions:**

A magnitude of the prevalence of underreporting *Leishmania* infection among Thai patients with HIV was revealed in this study. Effective public health policy to prevent and control disease transmission is urgently needed.

## Author summary

Visceral leishmaniasis (VL) in Thailand is caused by two causative agents, *Leishmania martiniquensis* and *Leishmania siamensis*. A public health concern brought us to investigate the magnitude of *Leishmania* infection among individuals with HIV living in an affected area, Trang province, southern Thailand. The results showed a high seroprevalence of *Leishmania* infection. Using PCR-based technique, DNA detection in the buffy coat was 8.4%, and 1.8% were symptomatic VL. Asymptomatic *Leishmania* infection could play an important role in disease transmission. Risk factors associated with *Leishmania* infection among Thai patients with HIV were firstly described. Those who were NIDU and lived in stilt houses were associated with *Leishmania* infection. Individuals who had lower immunity with detectable viral load were more likely to contract the infection. Interestingly, not only *L*. *martiniquensis* and *L*. *siamensis* were identified but also *L*. *donovani* complex, *L*. *major*, and *L*. *lainsoni*, were firstly reported among indigenous Thai people. These findings could lead to effective intervention and prevention methods to control leishmaniasis in Thailand. Further studies are needed to investigate the disease development in asymptomatic infections as well as evaluate the prevalence in other regions of Thailand.

## Introduction

Co-infection of leishmaniasis and human immunodeficiency virus (HIV*)* is a major public health problem globally. *Leishmania* and HIV each promote the activation of the other, causing host immune impairment. The co-infection results in treatment failure, high relapse, and high mortality rate [1]. Meta-analysis has revealed that the direct agglutination test (DAT) gave high sensitivity and specificity for serodiagnosis of VL when compared to other serological tests [2]. However, low sensitivity of serological tests for VL diagnosis has been shown among these patients due to defective host immunity [3]. To increase the sensitivity of *Leishmania* DNA detection, the polymerase chain reaction (PCR) using blood samples has been suggested [4]. The internal transcribed spacer 1 (ITS1)-PCR method has been recommended to detect *Leishmania* DNA [5].

In Thailand, the first autochthonous VL case was documented in a 3-year-old girl living in a southern province in 1999 [6]. Until 2012, *L*. *siamensis* was firstly reported in a patient with HIV in Trang province. Since then, CL and/or VL have been sporadically reported in immunocompetent and immunocompromised patients predominantly in the south and north of Thailand and about 40% were patients with HIV/AIDS. *L*. *martiniquensis* was the predominant causative agent while *L*. *siamensis* was indigenously reported in only one Thai patient [7].

Information of the true prevalence of *Leishmania* infection among Thai patients with HIV, a high risk group, is still lacking. Thus, the objectives of this study were to determine the prevalence and the risk factors associated with *Leishmania* infection among patients with HIV/AIDS in Trang province, southern Thailand.

## Methods

### Study design and population

A cross-sectional study of *Leishmania* infection was conducted between February 2015 and February 2016. Eligible participants were >18 years old and attending an HIV clinic, Trang Hospital, Trang province. They visited the clinic every 6 months for follow-up testing and to receive antiretroviral therapy (ART). They lived in ten districts of Trang province, other nine provinces located in the south, and three provinces in other regions of Thailand. Clinical information of participants was collected from patients’ medical records.

### Ethics statement

Written informed consent was obtained from all participants. All participants were >18 years old. All analyzed data were anonymized. The research protocol was approved by the Ethics Committee of the Royal Thai Army Medical Department and the Ethics Committee of Mahidol University, Thailand.

### Blood collection

Eight milliliters of EDTA anti-coagulated blood samples were collected. The whole blood was centrifuged at 900 × *g* for 10 minutes to separate the plasma and buffy coat and was then kept at −20°C until further use.

### Definition

Seropositivity of *Leishmania* infection was defined as detection of antibodies in individuals who were exposed to *Leishmania* infection and being either symptomatic or asymptomatic.

Asymptomatic *Leishmania* infection was defined as individuals who experienced no symptoms of VL but presented a positive test by DAT or PCR assays.

Symptomatic VL was defined as individuals having a history of fever lasting at least 2 weeks with splenomegaly. One or more of the following clinical characteristics may be observed: hepatomegaly, weight loss, anemia, leucopenia, thrombocytopenia, and hypergammaglobulinemia. Detection of the parasites must be confirmed under microscopic examination or by PCR assay using any clinical samples (e.g., bone marrow aspirates, lymph node, blood, and/or other biopsy samples).

### Detection of *Leishmania* antibodies

*Leishmania* antibodies were assayed using the commercial DAT kit (Biomedical Research) according to the manufacturer’s instruction. The positive plasma control was obtained from confirmed VL cases using the PCR method. For the negative control, plasma from healthy individuals was used. The cutoff value of positive DAT titers was ≥1:100 following manufacturer recommendation.

### *Leishmania* DNA detection

DNA was extracted from 200 μL of buffy coat sample using Gen UP gDNA Kit (Biotech). Nested PCR was used to amplify the ITS1 region of the ribosomal DNA (rDNA) gene of *Leishmania*. In the primary PCR, primers LITSR and L5.8S were used to amplify the 319–348 amplicons [8]. The newly designed secondary primers LITSR2 (CTG-GAT-CAT-TTT-CCG-ATG-ATT) and L5.8S inner (GTT-ATG-TGA-GCC-GTT-ATC-C) generated 230–280 amplicons depending on *Leishmania* species. PCR reactions were performed using the MJ Mini thermal cycler (BioRad) in volumes of 25 μL, containing 12.5 pmol of each primer, 0.2 mM dNTP, 1.5 mM MgCl_2_, 1× PCR buffer, 1 U of *Taq* DNA polymerase, and 4 μL of DNA template. DNA of *L*. *martiniquensis* promastigotes (MHOM/MQ/92/MAR1) was used as the positive control. The condition was started by pre-denaturation at 94°C for 3 minutes followed by 35 cycles: denaturation at 94°C for 1 minute, annealing temperature at 54°C for 30 seconds, and extension at 72°C for 30 seconds. Final extension was at 72°C for 5 minutes. PCR products were separated by electrophoresis in 1.5% agarose gel stained with SYBR Safe (Invitrogen). The results were visualized and documented by Molecular Imager Gel Doc XR+ System with Imager Lab 3.0 (BioRad).

### Sequence analysis

Positive PCR products were sent to U2Bio Co. Ltd., South Korea for sequencing. Chromatograms were validated using BioEdit version 7.0.1. The sequences were multiple-aligned with reference *Leishmania* strains retrieved from GenBank. The phylogenetic tree was constructed by using the neighbor-joining (NJ) method using the MEGA program, version 7.0. The reliability was tested by 1,000 bootstrap replications and Tajima-Nei was selected for the DNA substitution model of phylogenetic analysis.

### Questionnaires

To determine the risk factors and outcomes of *Leishmania* infection, standardized questionnaires were used. Enrolled subjects with HIV were interviewed face-to-face covering demographic data, socioeconomic status, clinical symptoms, and associated risk behaviors.

### Statistical analysis

The association between potential risk factors and *Leishmania* infection was assessed by univariate and multivariate logistic regression analysis. Odds ratios and 95% confidence intervals (CI) were calculated and *p* values <0.05 were considered statistically significant. All analyses were performed using STATA, version SE14 (Stata Corporation, College Station, TX, USA). (http://dx.doi.org/10.17504/protocols.io.j2dcqa6)

## Results

### Study population and characteristics

A total of 724 participants with HIV were enrolled in this study. Of these, 643 (88.8%) filled out questionnaires. Living areas of participants were as follows: 570 (88.6%) lived in Trang province and 67 (10.4%) lived in nine other provinces located in the south. Only five (0.8%) were from other regions of Thailand. The mean age was 43.6 ± 8.5 years. The characteristics of the enrolled subjects are shown in Table 1. Their clinical characteristics and risk behaviors during the past one year (S1 and S2 Tables) included history of injection drug users (IDUs) at 16.6% while 13.7% were non-injection drug users (NIDUs). A total of 512 (79.6%) subjects lived in non-stilt houses while 131 (20.4%) lived in stilt houses. Most of the individuals (68.6%) had CD4^+^ levels more than 500 cells/μL and only 9.6% were less than 200 cells/μL.

**Table 1:** The characteristics of enrolled patients with HIV (n = 643) were analyzed using three categories: i) patients who were either seropositive by DAT analysis with titers of >100 or positive by PCR assay; ii) patients who were seropositive by DAT analysis with titers of >100; and iii) patients who were positive only by PCR assay.

Characteristics	Total examined	No. of positive DAT or PCR (%)	*p-value*	No. of positive DAT (%)	*p-value*	No. of positive PCR (%)	*p-value*
Age (mean ± SD)	43.6 ± 8.5	43.1 ± 8.4	0.40	42.6 **±** 0.8	0.17	44.7 **±** 9.4	0.28
Gender							
Male	331 (51.5)	81 (24.5)	0.54	59 (17.8)	0.57	29 (8.8)	0.85
Female	312 (48.5)	83 (26.6)		61 (19.6)		26 (8.3)	
Educational level							
Primary school	227 (35.3)	69 (24.9)	0.28	51 (18.4)	0.12	22 (7.9)	0.74
Secondary school	199 (30.9)	45 (22.6)		29 (14.6)		18 (9.1)	
Vocational school	80 (12.4)	27 (33.8)		21 (26.3)		9 (11.3)	
Bachelor and graduated	87 (13.5)	23 (26.4)		19 (21.8)		6 (6.9)	
Occupation							
Unemployed	58 (9.0)	15 (25.9)	0.98	12 (20.7)	0.75	5 (8.6)	0.98
Agriculture	197 (30.6)	49 (24.9)		35 (17.8)		19 (9.6)	
Government	48 (7.5)	13 (27.1)		11 (22.9)		4 (8.3)	
Business	130 (20.2)	36 (27.7)		27 (20.8)		10 (7.7)	
Laborer	147 (22.9)	37 (25.2)		27 (18.4)		11 (7.5)	
Others	63 (9.8)	14 (22.2)		8 (12.7)		6 (9.5)	
Living areas							
Trang Province	570 (88.6)	147 (25.8)		105 (18.4)		50 (8.8)	
Other 9 provinces located in the south	67 (10.4)	17 (25.4)		15 (22.4)		5 (7.5)	
Central part	5 (0.8)	0		0		0	
Other parts	1 (0.2)	0		0		0	

### Prevalence of *Leishmania* infection

Three categories of data analysis of the prevalence of *Leishmania* infection were performed (Table 1). The first group comprised patients who were either seropositive by DAT analysis with titers of ≥100 or positive by PCR assay. The second category involved patients who were seropositive by DAT analysis with titers of ≥100, and the last group comprised patients who were positive only by PCR assay. The prevalence of *Leishmania* infection using positive results either by DAT or PCR assays was 25.1% (182/724). Seropositive cases comprised 18.5% (134/724) while *Leishmania* DNA detection by PCR was 8.4% (61/724). Only 1.8% (13/724) were positive using both methods (Table 2). Table 3 shows numbers of positive *Leishmania* infection using DAT, the titer of DAT, and PCR. Thus, the overall prevalence of asymptomatic *Leishmania* infection was 24.9 (180/724). Tables 4 and 5 show affected areas of *Leishmania* infections.

**Table 2:** Number of *Leishmania* infections by DAT analysis with titers of ≥100 or by PCR assay (n = 724).

Diagnostic Method		PCR assay		Total number
		Number positive	Number negative	
**DAT analysis**	**Number positive**	13	121	134
	**Number negative**	48	542	590
**Total number**		61	663	724

**Table 3:** Numbers of positive DAT analysis together with numbers of positive PCR results at the titers of 1:100 to 1:6400.

DAT (Antibody titer)	No. of positive DAT	No. of positive PCR
1:100	13	1
1:200	15	2
1:400	52	3
1:800	34	3
1:1600	18	2
1:3200	0	0
1:6400	2	2

**Table 4:** Patients who were either seropositive by DAT analysis with titers of ≥100 or positive by PCR assay among 13 provinces.

Part of Thailand	Province	No. of positive cases	No. of participants
-	No data	0	1
Central	Bangkok	0	3
	Nonthaburi	0	1
	Prachuap Khiri Khan	0	1
South	Chumphon	0	1
	Krabi	9	24
	Nakhon Si Thammarat	1	9
	Phang-Nga	0	3
	Phatthalung	2	8
	Phuket	1	5
	Satun	1	4
	Songkhla	2	8
	Surat Thani	1	5
	Trang	147	570
Total		164	643

**Table 5:** Patients who were either seropositive by DAT analysis with titers of ≥100 or positive by PCR assay among 10 districts of Trang province.

Ten districts of Trang Province	No. of positive cases	No. of participants
Had Someran	1	5
Huai Yot	8	48
Kantang	21	72
Meuang	89	332
Nayong	7	30
Palien	5	17
Ratsada	2	7
Sikao	7	20
Wang Wiset	5	21
Yantakao	2	17
No data	0	1
	147	570

**Fig 1:**
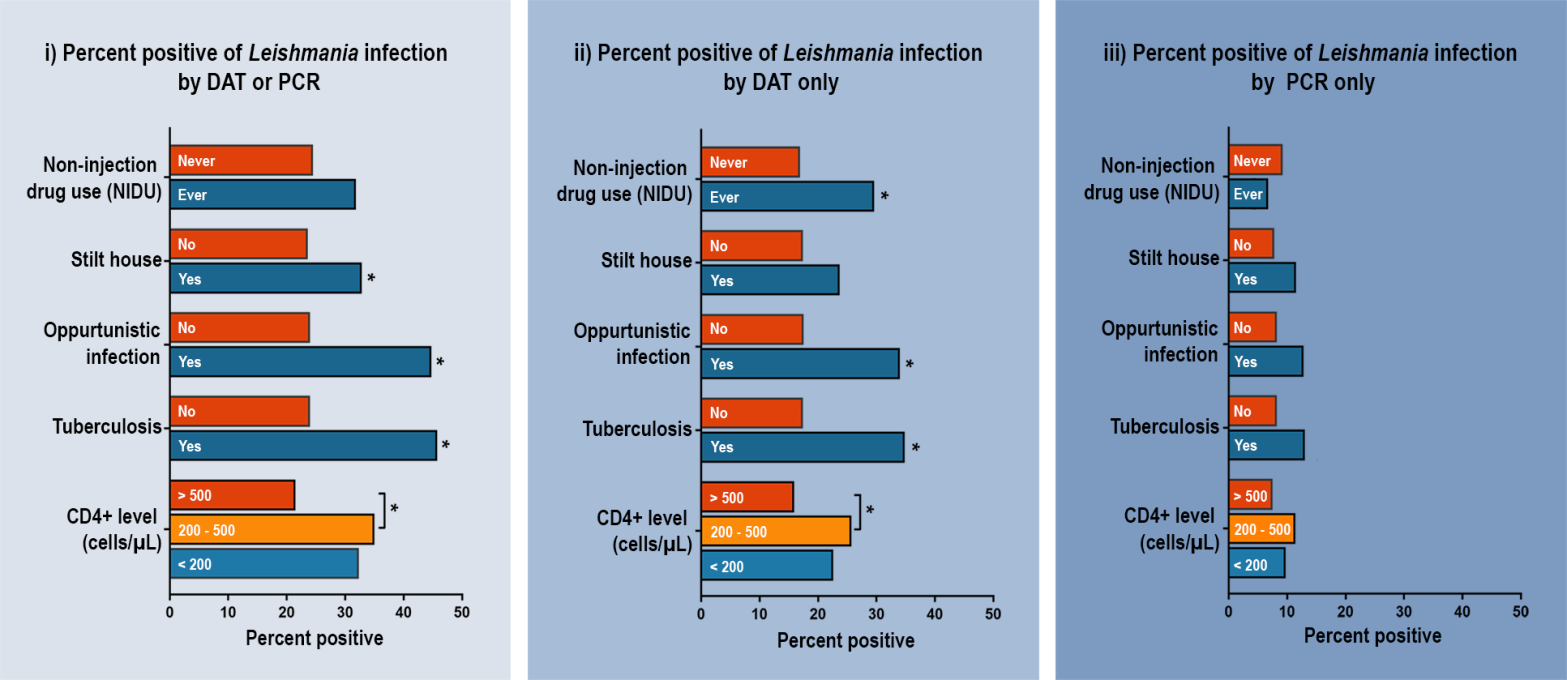
The percent of positive *Leishmania* infections by clinical characteristics and risk behaviors of enrolled patients with HIV (n = 643) was characterized into three categories: i) positivity diagnosed by DAT analysis with titers of ≥100 or by PCR assay, ii) positivity diagnosed by DAT analysis with titers of ≥100, and iii) positivity diagnosed by PCR assay.

Regarding the analysis of those who were either seropositive by DAT or positive by PCR assay, the prevalence of *Leishmania* infection significantly differed among participants who lived in stilt houses compared to those living in non-stilt houses (*p* = 0.03), those who developed jaundice (*p* = 0.02), having opportunistic infection (*p* = 0.002) especially tuberculosis (*p* = 0.001), and those having low CD4^+^ levels <500 cells/μL (*p* = 0.003) (Fig 1). No significant difference was found among age group, gender, educational level, occupation, working outdoors at night, average income, years of HIV diagnosis, viral load, history of going abroad, drug use (IDUs/NIDUs), pet/animal owner, animal shed nearby the house, plantation nearby the house, and bed net use (S1 and S2 Tables).

### Symptomatic VL

Two patients showed symptomatic VL. The first case was a 39-year-old herdsman living in Phuket province. He had a history of NIDU. He presented with a fever for more than two weeks. Many nodules were observed on the trunk. Laboratory findings revealed CD4^+^ levels at 173 cells/μL with an undetectable viral load. The DAT titer was 1:6400. The causative agent was *L*. *martiniquensis*, which was identified by the nested ITS1-PCR using the buffy coat and skin biopsy. The patient was treated with amphotericin B. However, he died of disease progression one year after initial VL diagnosis. The second case involved a 41-year-old male who originally lived in Trang province. He worked on a rubber plantation. He was both an IDU and NIDU. He developed epistaxis and bleeding gums. Laboratory findings revealed pancytopenia and CD4^+^ levels of 622 cells/μL with an undetectable viral load. Intracellular amastigotes were observed from the lymph node biopsy with Giemsa stain. The DAT titer was 1:6400 and the causative agent was *L*. *martiniquensis*, which was identified using the nested ITS1-PCR of the buffy coat. The patient was treated with amphotericin B but he died within one week after treatment.

### Risk factor analysis

Univariate and multivariate analysis of risk factors for acquiring *Leishmania* infections by DAT titers of ≥100 or positive by PCR assays are shown in Table 6. Univariate analysis showed that participants who lived in stilt houses had higher risk (OR = 1.58, 95% CI = 1.04–2.39) of contracting *Leishmania* when compared with those living in non-stilt houses. CD4^+^ levels between 200 and 500 cells/μL were at higher risk than those who had CD4^+^ levels >500 cells/μL (OR = 1.70, 95% CI = 1.29–2.97). After adjusting for age, gender, NIDUs, history of traveling abroad, pet owners and raising animals in housing areas, bed net use, animal shed and plantation nearby the house, underlying diseases, viral load, and duration of HIV diagnosis, multivariate logistic regression analysis revealed that those living in stilt houses had greater risk (OR = 1.60, 95% CI = 1.04–2.47) of acquiring the infection when compared with those living in non-stilt houses. In addition, those who had CD4^+^ levels 200–500 cells/μL (OR = 2.13, 95% CI = 1.36–3.32) and <200 cells/μL (OR = 1.98, 95% CI = 1.06–3.73) had higher risk of contracting *Leishmania* than those who had CD4^+^ levels >500 cells/μL. In addition, multivariate analysis of the associated risk factors of *Leishmania* infection using only seropositivity showed that participants who were NIDU had higher risk of presenting *Leishmania* seropositivity than those who were not (OR = 2.23, 95% CI = 1.27–3.92). In addition, those who had CD4^+^ levels 200–500 cells/μL were also at higher risk of being seropositive than those who had CD4^+^ levels >500 cells/μL (OR = 2.09, 95% CI = 1.27–3.44) (S3 Table). Multivariate analysis of the associated risk factors of *Leishmania* infection using only positive PCR results showed that those who had a detectable viral load >50 copies/mL were at higher risk of acquiring detectable *Leishmania* DNA in the blood (OR = 2.31, 95% CI = 1.01–5.29) than those who had an undetectable viral load after adjusting for those variables as mentioned above (S4 Table).

**Table 6:** Univariate and multivariate analysis of associated risk factors of *Leishmania* infection using seropositive results by DAT analysis with titers of ≥100 or positive by PCR assay.

Characteristics	Crude Odds Ratio	95% CI	*p*-value	Adjusted Odds Ratio	95% CI	*p*-value
Age	0.99	0.97–1.01	0.40	0.99	0.97–1.01	0.41
Gender						
Male	1.00			1.00		
Female	1.12	0.78–1.60	0.54	1.29	0.87–1.90	0.21
Non-injection drug users (NIDUs)						
Never	1.00			1.00		
Ever	1.44	0.88–2.34	0.15	1.49	0.87–2.55	0.14
History of going abroad						
No	1.00			1.00		
Yes	0.85	0.49–1.47	0.56	0.86	0.49–1.52	0.60
Pet owner						
No	1.00			1.00		
Yes	0.98	0.69–1.40	0.91	1.00	0.68–1.45	0.99
Raising animals						
No	1.00			1.00		
Yes	0.74	0.47–1.16	0.19	0.79	0.48–1.30	0.35
Stilt house						
No	1.00			1.00		
Yes	1.58	1.04–2.39	**0.030**	1.57	1.02–2.41	**0.042**
Animal shed near the house						
No	1.00			1.00		
Yes	1.07	0.62–1.81	0.15	1.19	0.65–2.18	0.57
Plantation near the house						
No	1.00			1.00		
Yes	0.71	0.45–1.13	0.15	0.66	0.39–1.10	0.11
Bed net use						
No	1.00			1.00		
Yes	0.86	0.60–1.23	0.40	0.88	0.60–1.27	0.49
Underlying disease						
No	1.00			1.00		
Yes	1.30	0.87–1.95	0.21	1.46	0.95–2.23	0.08
CD4+ (cells/μL)						
>500	1.00			1.00		
200–500	1.96	1.29–2.97	**0.001**	2.17	1.38–3.41	**0.001**
<200	1.73	0.97–3.09	0.06	1.97	1.05–3.69	**0.036**
Viral load						
Undetectable (<50 copies/mL)	1.00			1.00		
Detectable	1.42	0.83–2.45	0.20	1.11	0.61–2.02	0.72
Duration of HIV diagnosis						
<5 years	1.00			1.00		
5–10 years	1.17	0.77–1.80	0.46	1.34	0.85–2.12	0.20
>10 years	1.19	0.74–1.92	0.47	1.49	0.90–2.48	0.12

### Phylogenetic analysis and *Leishmania* species identification

**Fig 2:**
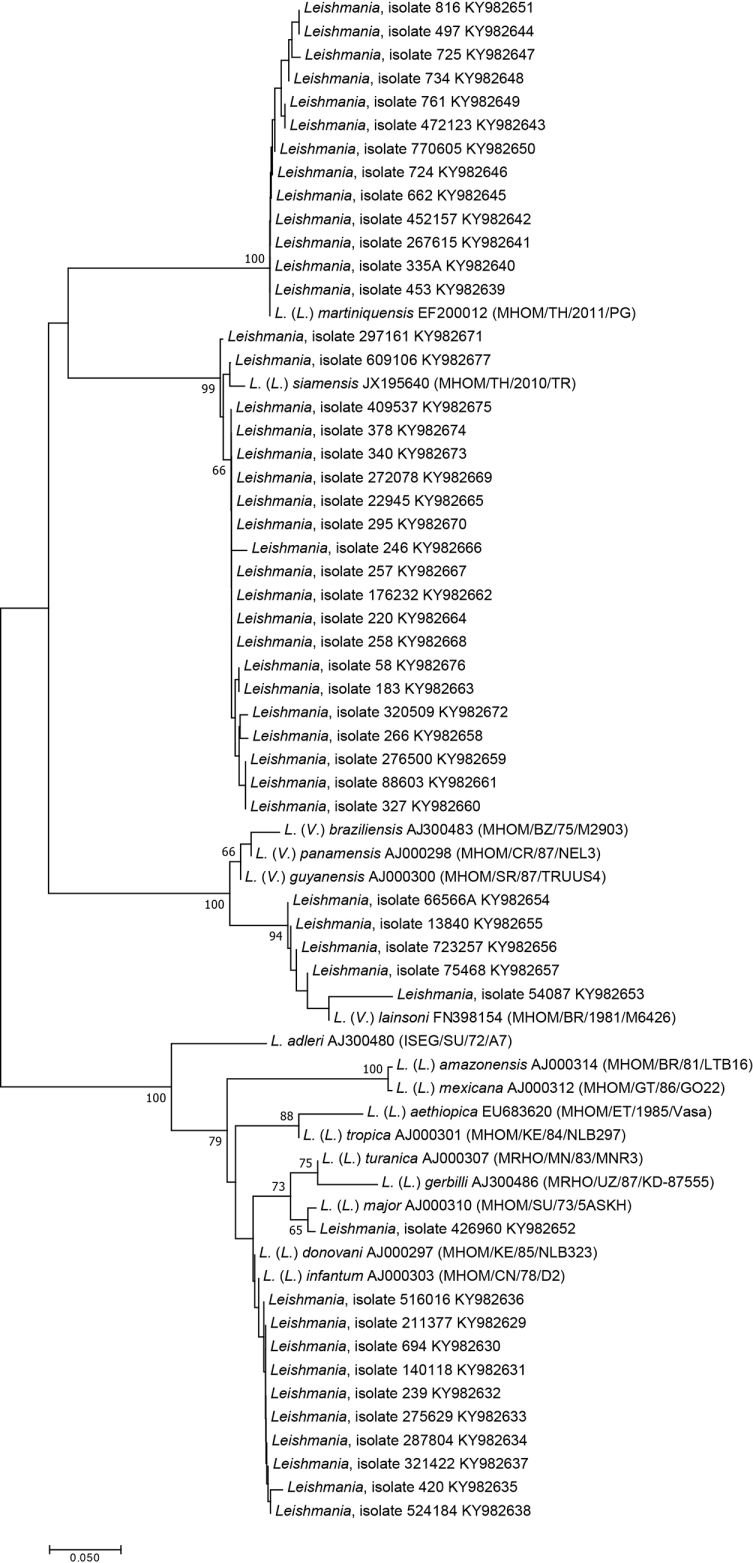
The unrooted phylogenetic tree inferred from the ITS1 sequences using the NJ method. The bootstrapping values < 50 are omitted. Dense lines indicate *Leishmania* species complexes.

Of 61 samples, nucleotide sequencing was successful for 49. These sequences, together with 16 reference sequences of different *Leishmania* species, were included to construct the phylogenetic tree using the NJ method (Fig 2). The phylogenetic analyses grouped the sequences into five separated clades. The majority of the samples (20 (40.8%) and 13 (26.5%)) were closely related to *L*. *siamensis* and *L*. *martiniquensis*, respectively, whereas ten (20.4%) were closely related to *L*. *donovani* complex, five (10.2%) were related to *L*. *lainsoni*, and one (2.1%) was related to *L*. *major*.

## Discussion

This was the first study providing important information of the prevalence of co-infection of *Leishmania* among Thai patients with HIV who had been regularly attending the HIV clinic in Trang province. The prevalence of *Leishmania* infection was approximately one-fourth of the 724 participants determined by either DAT or PCR assays. In the past, *Leishmania* infections were previously reported in five provinces in the south (Surat Thani [6], Phang-Nga [9], Trang [10], Songkhla [11], and Satun [12]) where most people mainly earn their living in agricultural sectors. The climate and humidity in the south are suitable for the sand fly’s habitat where potential sand fly vectors have been reported [13, 14]. Our study revealed four new affected areas of *Leishmania* infections in the south (Phuket, Krabi, Nakhon Si Thammarat, and Phatthalung provinces). Thus, at present, overall affected areas cover nine southern provinces.

For VL/HIV co-infection, the sensitivities of DAT to detect antibodies against *Leishmania* infection were 50–84% [15]. Related studies of VL in immunocompetent subjects in endemic areas in India and Iran used DAT titers at different cutoff values ranging from 1:800 to 1:3200 [16–18]. A cutoff value at a titer of 1:100 was previously used to screen VL among HIV-positive patients who developed clinical symptoms in northeast Iran [19]. A low DAT titer of 1:200 was also detected in an immunocompetent VL Thai patient caused by *L*. *martiniquensis* [9]. In this study, one patient having a DAT titer of 1:100 also produced a positive PCR result. Therefore, a positive serological test at low titers could have diagnostic value to detect the infection. Thus, the cutoff values of DAT varied when conducted in different study populations as well as areas of study where cross-reactivity of DAT against other blood parasite infections could have occurred. A systematic review revealed that DAT titers detected in symptomatic patients were higher than those of asymptomatic patients [20]. Patients presenting very high DAT titer would have significantly greater disease progression than those presenting low titers [21]. In this study, clinical characteristics of VL were observed in two symptomatic cases that had DAT titers of 1:6400 together with positive PCR results. Thus, a close follow-up for those asymptomatic infections showing positive results of DAT or PCR is needed.

In this study, serological and molecular diagnosis among individuals with HIV was not in concordance with other studies [22]. Our results showed that PCR positivity was low when compared with numbers of DAT positivity. The potential reasons among DAT positive individuals who might become PCR negative could include degradation and clearance of *Leishmania* DNA after infection, which corresponds to development of protective immunity due to the use of antiviral drugs for HIV. A positive PCR test among DAT negative individuals could occur when the individual was bitten by a *Leishmania* infected sand fly, but either immunity has not yet developed or antibody levels are too low to be detected by the methods employed, especially in HIV-positive populations [23].

Before using HAART, asymptomatic infection in HIV-*Leishmania* co-infection in Europe was 4–33% [24]. However, the incidence has been reduced to 20% after ART drugs had been given to all individuals with HIV [3]. In this study, all patients with HIV regularly received HAART treatment that could restore TH1 cytokine and antibody production [25]. HAART-treated HIV patients demonstrated a better ability to control *Leishmania* infection [26]. Many patients with subclinical VL did not develop clinical symptoms after taking HAART medications while some developed the disease [3]. Other risk factors (e.g., stage of HIV infection, parasite virulence, drug resistance, nutritional status, age, and gender) may be involved in disease progression [1, 27]. Prospective studies are needed to determine disease progression in this population.

In this study, most enrolled participants (88.6%) were not randomly selected and originally lived in Trang province. Thus, our results do not represent the prevalence of co-infection of *Leishmania* and patients with HIV of the country, and they do not represent the prevalence in each district of Trang province. However, this study showed the magnitude of the seroprevalence and significant numbers of subclinical results of *Leishmania* DNA detection circulating in the blood in Thai individuals with HIV.

In southern Europe, IDUs were the most important risk factor accounting for more than 90% of all cases [28]. Our results showed no significant difference in prevalence among IDUs, while seroprevalence was associated with NIDUs. Leishmaniasis was also associated with socioeconomic status. In India, housing materials such as mud, plants, and earthen floors were risk factors for VL [20, 29]. Our study showed that living in stilt houses was an independent associated risk factor for *Leishmania* infection. The presence of stilts provided an open area under the house that might have increased chances of sand fly bites to humans as well as providing resting sites for sand flies. Sand flies frequently bite at dusk [30] when most people spend their time at the open area of the house. In addition, CD4^+^ levels play an important role to protect the host from opportunistic infections. Related studies showed that the first episode of symptomatic VL diagnosis involved more than 80% of patients with HIV who had low CD4^+^ levels [26, 31]. Our results also confirmed that low CD4^+^ levels (<200 cells/μL) as well as 200 to 500 cells/μL significantly increased the risk of *Leishmania* infection.

In this study, *Leishmania* DNA detection by PCR assay was associated with detectable viral load. No correlation has been reported between PCR positivity and CD4^+^ levels, whereas the correlation between HIV viral load and parasitemia was observed among asymptomatic patients [22]. Clinical progression of HIV/AIDS was simultaneously promoted by VL. HIV infection enhances parasite growth by modulating significant cytokine response to *Leishmania* while the parasite upregulates viral expression [3].

Public health awareness of *Leishmania* infection in Thailand started when one autochthonous VL was reported in 1996. However, at that time, the disease was uncommon as well as unfamiliar to most physicians, which could have led to a lot of underreporting of leishmaniasis cases in the past 20 years. Using the molecular method, this cross-sectional study was the first to systematically estimate the prevalence of *Leishmania* infection among patients with HIV, a high risk group, revealing not only *L*. *siamensis* and *L*. *martiniquensis* infection but also infections of other species (e.g., *L*. *donovani* complex, *L*. *lainsoni*, and *L*. *major*) that already existed in the affected area. From the phylogenetic analysis, the ITS1 region is one of the more powerful targets used to discriminate the *Leishmania* species [32]. Our previous study showed that the ITS1 region had the highest sensitivity to detect *L*. *martiniquensis* and *L*. *siamensis* compared with the other genes: *hsp70*, *cyt* b, and SSU-rRNA [5]. Additionally, we used *hsp70*-PCR and kDNA-PCR to amplify DNA samples of *L*. *major*, *L*. *donovani*, *L*. *lainsoni*, *L*. *siamensis*, and *L*. *martiniquenensis*. Unfortunately, negative PCR results were obtained due to lower sensitivities of PCR amplification using these genes.

*L*. *martiniquensis* and *L*. *siamensis* were the predominant species detected in this study. Additionally, *L*. *infantum* (which causes VL) was previously reported in an HIV negative individual living in Bangkok [33]. The *L*. *donovani* complex species are the major causative agents of VL worldwide. The distribution of the complex species might have been introduced by travelers or workers from VL-endemic areas to Thailand [34]. *L*. *major*, a causative agent for CL in the Old World, causes zoonotic transmission, especially Afghanistan and India [31]. VL, caused by *L*. *major*, has occasionally been reported among patients with HIV [3]. *L*. *donovani* complex species and *L*. *major* have been reported in China, Bangladesh, India, and Nepal [15]. Thus, distribution of these two species in Thailand could be possible. *L*. *lainsoni* infection causes localized CL and was reported in South America in Bolivia, Peru, Suriname, French Guiana, and Brazil [15, 35]. This is the first report of *L*. *major* and *L*. *lainsoni* infection among individuals with HIV in Thailand. In addition, no report of *L*. *lainsoni* has been documented in the Old World, especially among patients with HIV.

To prevent and control VL, understanding disease epidemiology is extremely important. A cohort study conducted in this population as well as studies of potential vectors and animal reservoirs are needed. Moreover, large scale molecular epidemiological studies in other high morbidity areas are required for this emerging disease.

## Supporting information

S1 Checklist STROBE checklist for cross-sectional studies. (DOCX)

S1 Table Clinical characteristics in the past year of enrolled patients with HIV (n = 643) were analyzed using three categories: i) patients who were either seropositive by DAT analysis with titers of >100 or positive by PCR assay, ii) patients who were seropositive by DAT analysis with titers of >100, and iii) patients who were positive only by PCR assay.
(DOCX)

S2 Table Characteristics of risk behaviors of enrolled patients with HIV (n = 643) were analyzed using three categories: i) patients who were either seropositive by DAT analysis with titers of >100 or positive by PCR assay, ii) patients who were seropositive by DAT analysis with titers of >100, and iii) patients who were positive only by PCR assay.
(DOCX)

S3 Table Univariate and multivariate analysis of associated risk factors of *Leishmania* infection using seropositive results by DAT analysis with titers of >100. (DOCX)

S4 Table Univariate and multivariate analysis of associated risk factors of *Leishmania* infection using seropositive results by PCR assay. (DOCX)
