# Supplementary material for: Prevalence and risk factors associated with Leishmania infection in Trang Province, southern Thailand
Source: PLoS Negl Trop Dis. 2017 Nov 20;11(11):e0006095. doi: 10.1371/journal.pntd.0006095 (PMC5714378; doi:10.1371/journal.pntd.0006095)
Supplement: S1 Table — Clinical characteristics in the past year of enrolled patients with HIV (n = 643) were analyzed using three categories: i) patients who were either seropositive by DAT analysis with titers of >100 or positive by PCR assay, ii) patients who were seropositive by DAT analysis with titers of >100, and iii) patients who were positive only by PCR assay. (DOCX) [file pntd.0006095.s002.docx]

**Supporting information**

**S1 Table : Clinical characteristics in the past one year of enrolled patients with HIV (n=643) were analyzed using three categories: i) patients who were either seropositive by DAT analysis with titers of >100 or positive by PCR assay; ii) patients who were seropositive by DAT analysis with titers of >100 and iii) patients who were positive only by PCR assay.**

| **Characteristics** | **Total examined** | | **No. of positive DAT or PCR (%)** | | ***p-value*** | | **No. of positive DAT (%)** | | ***p-value*** | | **No. of positive PCR (%)** | | ***p-value*** | |  |
| --- | --- | --- | --- | --- | --- | --- | --- | --- | --- | --- | --- | --- | --- | --- | --- |
| Duration of HIV diagnosis | |  | |  | |  | |  | |  | |  | |  | |
| < 5 years | | 197 (30.6) | | 46 (23.4) | | 0.71 | | 34 (17.3) | | 0.80 | | 13 (6.6) | | 0.43 | |
| 5 – 10 years | | 281 (43.7) | | 74 (26.3) | |  | | 53 (18.9) | |  | | 28 (10.0) | |  | |
| > 10 years | | 165 (25.7) | | 44 (26.7) | |  | | 33 (20.0) | |  | | 14 (8.5) | |  | |
| CD4+ (cells/µL) | |  | |  | |  | |  | |  | |  | |  | |
| >500 | | 441 (68.6) | | 95 (21.5) | | **0.003*** | | 70 (15.9) | | **0.024*** | | 33 (7.5) | | 0.33 | |
| 200 – 500 | | 140 (21.8) | | 49 (35.0) | |  | | 36 (25.7) | |  | | 16 (11.4) | |  | |
| < 200 | | 62 (9.6) | | 20 (32.3) | |  | | 14 (22.6) | |  | | 6 (9.7) | |  | |
| Viral load | |  | |  | |  | |  | |  | |  | |  | |
| Undetectable   (<50 copies/mL) | | 574 (89.3) | | 142 (24.7) | | 0.20 | | 107 (18.6) | | 0.97 | | 45 (7.8) | | 0.06 | |
| Detectable | | 69 (10.7) | | 22 (31.9) | |  | | 13 (18.8) | |  | | 10 (14.5) | |  | |
| Underlying disease | |  | |  | |  | |  | |  | |  | |  | |
| No | | 490 (76.2) | | 119 (24.3) | | 0.20 | | 87 (17.8) | | 0.29 | | 36 (7.4) | | 0.05 | |
| Yes | | 153 (23.8) | | 45 (29.4) | |  | | 33 (21.6) | |  | | 19 (12.4) | |  | |
| Hypertension | |  | |  | |  | |  | |  | |  | |  | |
| No | | 607 (94.4) | | 152 (25.0) | | 0.27 | | 112 (18.5) | | 0.57 | | 50 (8.2) | | 0.24 | |
| Yes | | 36 (5.6) | | 12 (33.3) | |  | | 8 (22.2) | |  | | 5 (13.9) | |  | |
| Dyslipidemia | |  | |  | |  | |  | |  | |  | |  | |
| No | | 561 (87.2) | | 140 (25.0) | | 0.40 | | 102 (18.2) | | 0.41 | | 44 (7.8) | | 0.09 | |
| Yes | | 82 (12.8) | | 24 (29.3) | |  | | 18 (22.0) | |  | | 11 (13.4) | |  | |
| Diabetes |  | |  | |  | |  | |  | | |  | |  |  |
| No | 632 (98.3) | | 160 (25.3) | | 0.41 | | 119 (18.8) | | 0.41 | | | 52 (8.2) | | **0.03*** |  |
| Yes | 11 (1.7) | | 4 (36.4) | |  | | 1 (9.1) | |  | | | 3 (27.3) | |  |  |
| Opportunistic infection |  | |  | |  | |  | |  | | |  | |  |  |
| No | 596 (92.7) | | 143 (24.0) | | **0.002*** | | 104 (17.5) | | **0.005*** | | | 49 (8.2) | | 0.28 |  |
| Yes | 47 (7.3) | | 21 (44.7) | |  | | 16 (34.0) | |  | | | 6 (12.8) | |  |  |
| Tuberculosis |  | |  | |  | |  | |  | | |  |  | |  |
| No | 597 (92.8) | | 143 (24.0) | | **0.001*** | | 104 (17.4) | | **0.004*** | | | 49 (8.2) | 0.26 | |  |
| Yes | 46 (7.2) | | 21 (45.7) | |  | | 16 (34.8) | |  | | | 6 (13.0) |  | |  |
| Fever |  | |  | |  | |  | |  | |  | |  | |  |
| No | 625 (97.2) | | 157 (25.1) | | 0.19 | | 115 (18.4) | | 0.31 | | 52 (8.3) | | 0.21 | |  |
| Yes | 18 (2.8) | | 7 (38.9) | |  | | 5 (27.8) | |  | | 3 (16.7) | |  | |  |
| Rash |  | |  | |  | |  | |  | |  | |  | |  |
| No | 632 (98.3) | | 161 (25.5) | | 0.89 | | 117 (18.5) | | 0.46 | | 55 (8.7) | | 0.31 | |  |
| Yes | 11 (1.7) | | 3 (27.3) | |  | | 3 (27.3) | |  | | 0 | |  | |  |
| Hyperpigmented skin |  | |  | |  | |  | |  | |  | |  | |  |
| No | 632 (98.3) | | 159 (25.2) | | 0.12 | | 117 (18.5) | | 0.46 | | 53 (8.4) | | 0.25 | |  |
| Yes | 11 (1.7) | | 5 (45.5) | |  | | 3 (27.3) | |  | | 2 (18.2) | |  | |  |
| Chronic lesion |  | |  | |  | |  | |  | |  | |  | |  |
| No | 634 (98.6) | | 160 (25.2) | | 0.19 | | 116 (18.3) | | **0.046*** | | 55 (8.7) | | 0.36 | |  |
| Yes | 9 (1.4) | | 4 (44.4) | |  | | 4 (44.4) | |  | | 0 | |  | |  |
| Nodule lesion |  | |  | |  | |  | |  | |  | |  | |  |
| No | 635 (98.8) | | 160 (25.2) | | 0.11 | | 117 (18.4) | | 0.17 | | 54 (8.5) | | 0.69 | |  |
| Yes | 8 (1.2) | | 4 (50.0) | |  | | 3 (37.5) | |  | | 1 (12.5) | |  | |  |
| Fatigue |  | |  | |  | |  | |  | |  | |  | |  |
| No | 641 (99.7) | | 164 (25.6) | | 0.41 | | 120 (18.7) | | 0.50 | | 55 (8.6) | | 0.67 | |  |
| Yes | 2 (0.3) | | 0 | |  | | 0 | |  | | 0 | |  | |  |
| Anorexia |  | |  | |  | |  | |  | |  | |  | |  |
| No | 639 (99.4) | | 163 (25.5) | | 0.98 | | 120 (18.8) | | 0.34 | | 54 (8.5) | | 0.24 | |  |
| Yes | 4 (0.6) | | 1 (25.0) | |  | | 0 | |  | | 1 (25.0) | |  | |  |
| Weight Loss |  | |  | |  | |  | |  | |  | |  | |  |
| No | 628 (97.7) | | 158 (25.2) | | 0.19 | | 114 (18.2) | | **0.030*** | | 54 (8.6) | | 0.79 | |  |
| Yes | 15 (2.3) | | 6 (40.0) | |  | | 6 (40.0) | |  | | 1 (6.7) | |  | |  |
| Bleeding gums |  | |  | |  | |  | |  | |  | |  | |  |
| No | 625 (97.2) | | 156 (25.0) | | 0.06 | | 114 (18.2) | | 0.11 | | 53 (8.5) | | 0.69 | |  |
| Yes | 18 (2.8) | | 8 (44.4) | |  | | 6 (33.3) | |  | | 2 (11.1) | |  | |  |
| Flatulence |  | |  | |  | |  | |  | |  | |  | |  |
| No | 623 (96.9) | | 157 (25.2) | | 0.32 | | 114 (18.3) | | 0.19 | | 54 (8.7) | | 0.56 | |  |
| Yes | 20 (3.1) | | 7 (35.0) | |  | | 6 (30.0) | |  | | 1 (5.0) | |  | |  |
| Jaundice |  | |  | |  | |  | |  | |  | |  | |  |
| No | 641 (99.7) | | 162 (25.3) | | **0.02*** | | 119 (18.6) | | 0.26 | | 54 (8.4) | | **0.04*** | |  |
| Yes | 2 (0.3) | | 2 (100.0) | |  | | 1 (50.0) | |  | | 1 (50.0) | |  | |  |
| Dyspnea |  | |  | |  | |  | |  | |  | |  | |  |
| No | 635 (98.8) | | 160 (25.2) | | 0.11 | | 116 (18.3) | | **0.022*** | | 55 (8.7) | | 0.38 | |  |
| Yes | 8 (1.2) | | 4 (50.0) | |  | | 4 (50.0) | |  | | 0 | |  | |  |

* *p* value < 0.05
