# Supplementary material for: Prevalence and risk factors associated with Leishmania infection in Trang Province, southern Thailand
Source: PLoS Negl Trop Dis. 2017 Nov 20;11(11):e0006095. doi: 10.1371/journal.pntd.0006095 (PMC5714378; doi:10.1371/journal.pntd.0006095)
Supplement: S2 Table — Characteristics of risk behaviors of enrolled patients with HIV (n = 643) were analyzed using three categories: i) patients who were either seropositive by DAT analysis with titers of >100 or positive by PCR assay, ii) patients who were seropositive by DAT analysis with titers of >100, and iii) patients who were positive only by PCR assay. (DOCX) [file pntd.0006095.s003.docx]

**Supporting information**

**S2 Table: Characteristics of risk behaviors of enrolled patients with HIV (n=643) were analyzed using three categories: i) patients who were either seropositive by DAT analysis with titers of >100 or positive by PCR assay; ii) patients who were seropositive by DAT analysis with titers of >100; and iii) patients who were positive only by PCR assay.**

| **Characteristics** | **Total examined** | **No. of positive DAT or PCR (%)** | ***p-value*** | **No. of positive DAT (%)** | ***p-value*** | **No. of positive PCR (%)** | ***p-value*** |
| --- | --- | --- | --- | --- | --- | --- | --- |
| Injection drug use (IDU) |  |  |  |  |  |  |  |
| Never | 536 (83.4) | 144 (26.9) | 0.08 | 109 (20.3) | **0.015*** | 45 (8.4) | 0.75 |
| Ever | 107 (16.6) | 20 (18.7) |  | 11 (10.3) |  | 10 (9.4) |  |
| Non- injection drug use (NIDU) |  |  |  |  |  |  |  |
| Never | 555 (86.3) | 136 (24.5) | 0.14 | 94 (16.9) | **0.005*** | 51 (9.2) | 0.15 |
| Ever | 88 (13.7) | 28 (31.8) |  | 26 (29.6) |  | 4 (4.6) |  |
| History of going abroad |  |  |  |  |  |  |  |
| No | 560 (87.1) | 145 (25.9) | 0.56 | 106 (18.9) | 0.65 | 50 (8.9) | 0.38 |
| Yes | 83 (12.9) | 19 (22.9) |  | 14 (16.9) |  | 5 (6.0) |  |
| Pet owner |  |  |  |  |  |  |  |
| No | 319 (49.6) | 82 (25.7) | 0.91 | 62 (19.4) | 0.62 | 25 (7.8) | 0.52 |
| Yes | 324 (50.4) | 82 (25.3) |  | 58 (17.9) |  | 30 (9.3) |  |
| Raising animals |  |  |  |  |  |  |  |
| No | 502 (78.1) | 134 (26.7) | 0.19 | 96 (19.1) | 0.57 | 46 (9.2) | 0.30 |
| Yes | 141 (21.9) | 30 (21.3) |  | 24 (17.0) |  | 9 (6.4) |  |
| Stilt house |  |  |  |  |  |  |  |
| No | 512 (79.6) | 121 (23.6) | **0.03*** | 89 (17.4) | 0.10 | 40 (7.8) | 0.18 |
| Yes | 131 (20.4) | 43 (32.8) |  | 31 (23.7) |  | 15 (11.5) |  |
| Animal shed nearby the house |  |  |  |  |  |  |  |
| No | 564 (87.7) | 143 (25.4) | 0.82 | 101 (17.9) | 0.19 | 52 (9.2) | 0.11 |
| Yes | 79 (12.3) | 21 (26.6) |  | 19 (24.1) |  | 3 (3.8) |  |
| Plantation nearby the house |  |  |  |  |  |  |  |
| No | 512 (79.6) | 137 (26.8) | 0.15 | 99 (19.3) | 0.39 | 47 (9.2) | 0.26 |
| Yes | 131 (20.4) | 27 (20.6) |  | 21 (16.0) |  | 8 (6.1) |  |
| Animal work at night |  |  |  |  |  |  |  |
| No | 599 (93.2) | 154 (25.7) | 0.66 | 110 (18.4) | 0.47 | 55 (9.2) | **0.04*** |
| Yes | 44 (6.8) | 10 (22.7) |  | 10 (22.7) |  | 0 |  |
| Plantation work at night |  |  |  |  |  |  |  |
| No | 365 (56.8) | 94 (25.8) | 0.87 | 69 (18.9) | 0.86 | 30 (8.2) | 0.73 |
| Yes | 278 (43.2) | 70 (25.2) |  | 51 (18.4) |  | 25 (9.0) |  |
| Fishing at night |  |  |  |  |  |  |  |
| No | 614 (95.5) | 154 (25.1) | 0.26 | 111 (18.1) | 0.08 | 30 (8.2) | 0.73 |
| Yes | 29 (4.5) | 10 (34.5) |  | 9 (31.0) |  | 25 (9.0) |  |
| Bed net use |  |  |  |  |  |  |  |
| No | 245 (38.1) | 67 (27.4) | 0.40 | 48 (19.6) | 0.64 | 22 (9.0) | 0.76 |
| Yes | 398 (61.9) | 97 (24.4) |  | 72 (18.1) |  | 33 (8.3) |  |

* *p* value < 0.05
