# Supplementary material for: Prevalence and risk factors associated with Leishmania infection in Trang Province, southern Thailand
Source: PLoS Negl Trop Dis. 2017 Nov 20;11(11):e0006095. doi: 10.1371/journal.pntd.0006095 (PMC5714378; doi:10.1371/journal.pntd.0006095)
Supplement: S3 Table — (DOCX) [file pntd.0006095.s004.docx]

**Supporting information**

**S3 Table: Univariate and multivariate analysis of associated risk factors of *Leishmania* infection using seropositive results by DAT analysis with titers of >100.**

| Characteristics | Crude Odd Ratio | 95% CI | *p*-value | Adjusted Odd Ration | 95% CI | *p*-value |
| --- | --- | --- | --- | --- | --- | --- |
| Age | 0.98 | 0.96-1.01 | 0.17 | 0.98 | 0.96-1.01 | 0.17 |
| Gender |  |  |  |  |  |  |
| Male | 1.00 |  |  | 1.00 |  |  |
| Female | 1.12 | 0.75-1.67 | 0.58 | 1.42 | 0.91-2.20 | 0.12 |
| Non-injection drug users (NIDUs) |  |  |  |  |  |  |
| Never | 1.00 |  |  | 1.00 |  |  |
| Ever | 2.06 | 1.24-3.42 | **0.005*** | 2.23 | 1.27-3.92 | **0.005*** |
| History of going abroad |  |  |  |  |  |  |
| No | 1.00 |  |  | 1.00 |  |  |
| Yes | 0.87 | 0.47-1.60 | 0.65 | 0.87 | 0.46-1.64 | 0.66 |
| Stilt house |  |  |  |  |  |  |
| No | 1.00 |  |  | 1.00 |  |  |
| Yes | 1.47 | 0.93-2.34 | 0.10 | 1.48 | 0.91-2.38 | 0.11 |
| Animal shed nearby the house |  |  |  |  |  |  |
| No | 1.00 |  |  | 1.00 |  |  |
| Yes | 1.45 | 0.83-2.54 | 0.19 | 1.41 | 0.77-2.58 | 0.27 |
| Plantation nearby the house |  |  |  |  |  |  |
| No | 1.00 |  |  | 1.00 |  |  |
| Yes | 1.45 | 0.83-2.54 | 0.19 | 1.41 | 0.77-2.58 | 0.27 |
| Bed net use |  |  |  |  |  |  |
| No | 1.00 |  |  | 1.00 |  |  |
| Yes | 0.91 | 0.60-1.36 | 0.64 | 0.94 | 0.62-1.44 | 0.79 |
| Underlying disease |  |  |  |  |  |  |
| No | 1.00 |  |  | 1.00 |  |  |
| Yes | 1.27 | 0.81-2.00 | 0.29 | 1.43 | 0.88-2.31 | 0.15 |
| CD4+ (cells/µL) |  |  |  |  |  |  |
| >500 | 1.00 |  |  | 1.00 |  |  |
| 200-500 | 1.83 | 1.16-2.90 | **0.009*** | 2.09 | 1.27-3.44 | **0.004*** |
| <200 | 1.55 | 0.81-2.95 | 0.19 | 1.90 | 0.94-3.85 | 0.07 |
| Viral load |  |  |  |  |  |  |
| Undetectable   (<50 copies/mL) | 1.00 |  |  | 1.00 |  |  |
| Detectable | 1.01 | 0.53-1.92 | 0.97 | 0.76 | 0.38-1.52 | 0.44 |
| Duration of HIV diagnosis |  |  |  |  |  |  |
| <5 years | 1.00 |  |  | 1.00 |  |  |
| 5 - 10 years | 1.11 | 0.69-1.79 | 0.66 | 1.22 | 0.73-2.03 | 0.45 |
| >10 years | 1.20 | 0.70-2.03 | 0.50 | 1.45 | 0.83-2.55 | 0.20 |

* *p* value < 0.05
