# Supplementary material for: Prevalence and risk factors associated with Leishmania infection in Trang Province, southern Thailand
Source: PLoS Negl Trop Dis. 2017 Nov 20;11(11):e0006095. doi: 10.1371/journal.pntd.0006095 (PMC5714378; doi:10.1371/journal.pntd.0006095)
Supplement: S4 Table — (DOCX) [file pntd.0006095.s005.docx]

**Supporting information**

**S4 Table: Univariate and multivariate analysis of associated risk factors of *Leishmania* infection using seropositive results by PCR assay.**

| Characteristics | Crude Odd Ratio | 95% CI | *p*-value | Adjusted Odd Ratio | 95% CI | *p*-value |
| --- | --- | --- | --- | --- | --- | --- |
| Age | 1.01 | 0.99-1.05 | 0.28 | 1.01 | 0.98-1.05 | 0.50 |
| Gender |  |  |  |  |  |  |
| Male | 1.00 |  |  | 1.00 |  |  |
| Female | 0.95 | 0.54-1.65 | 0.85 | 0.89 | 0.49-1.62 | 0.70 |
| Non-injection drug users (NIDUs) |  |  |  |  |  |  |
| Never | 1.00 |  |  | 1.00 |  |  |
| Ever | 0.47 | 0.17-1.34 | 0.16 | 0.45 | 0.15-1.32 | 0.15 |
| History of going abroad |  |  |  |  |  |  |
| No | 1.00 |  |  | 1.00 |  |  |
| Yes | 0.65 | 0.25-1.69 | 0.38 | 0.65 | 0.24-1.75 | 0.40 |
| Stilt house |  |  |  |  |  |  |
| No | 1.00 |  |  | 1.00 |  |  |
| Yes | 1.53 | 0.81-2.86 | 0.19 | 1.44 | 0.76-2.76 | 0.27 |
| Animal shed nearby the house |  |  |  |  |  |  |
| No | 1.00 |  |  | 1.00 |  |  |
| Yes | 0.39 | 0.12-1.28 | 0.12 | 0.48 | 0.14-1.66 | 0.25 |
| Plantation nearby the house |  |  |  |  |  |  |
| No | 1.00 |  |  | 1.00 |  |  |
| Yes | 0.64 | 0.30-1.40 | 0.27 | 0.70 | 0.31-1.57 | 0.39 |
| Bed net use |  |  |  |  |  |  |
| No | 1.00 |  |  | 1.00 |  |  |
| Yes | 0.92 | 0.52-1.61 | 0.76 | 0.87 | 0.49-1.56 | 0.64 |
| Underlying disease |  |  |  |  |  |  |
| No | 1.00 |  |  | 1.00 |  |  |
| Yes | 1.79 | 0.99-3.22 | 0.05 | 1.87 | 0.99-3.52 | 0.05 |
| CD4+ (cells/µL) |  |  |  |  |  |  |
| >500 | 1.00 |  |  | 1.00 |  |  |
| 200-500 | 1.60 | 0.85-3.00 | 0.15 | 1.71 | 0.86-3.41 | 0.13 |
| <200 | 1.32 | 0.53-3.30 | 0.55 | 1.21 | 0.45-3.25 | 0.71 |
| Viral load |  |  |  |  |  |  |
| Undetectable  (<50 copies/mL) | 1.00 |  |  | 1.00 |  |  |
| Detectable | 1.99 | 0.95-4.16 | 0.07 | 2.31 | 1.01-5.29 | **0.048*** |
| Duration of HIV diagnosis |  |  |  |  |  |  |
| <5 years | 1.00 |  |  | 1.00 |  |  |
| 5 - 10 years | 1.57 | 0.79-3.10 | 0.20 | 1.85 | 0.89-3.85 | 0.10 |
| >10 years | 1.31 | 0.60-2.88 | 0.50 | 1.55 | 0.68-3.54 | 0.30 |

* *p* value < 0.05
